# Right Thyroid Lobe Agenesis With Contralateral Compensatory Hypertrophy: A Case Report

**DOI:** 10.7759/cureus.105986

**Published:** 2026-03-27

**Authors:** Mauricio Aparicio Ramirez, César Adrian Gándara Diaz de Leon

**Affiliations:** 1 Medicine, Universidad Cuauhtemoc Campus Aguascalientes, Aguascalientes, MEX; 2 Internal Medicine, Hospital General Aguascalientes ISSSTE (Instituto de Seguridad y Servicios Sociales de los Trabajadores del Estado), Aguascalientes, MEX

**Keywords:** congenital abnormalities, goiter, hypertrophy, thyroid dysgenesis, thyroid gland, thyrotropin

## Abstract

Thyroid hemiagenesis is a rare congenital anomaly with a marked predominance of left lobe involvement. Right lobe agenesis is an exceptionally uncommon presentation that can pose significant diagnostic challenges. We report an unusual case of right thyroid lobe agenesis with contralateral compensatory hypertrophy in a 52-year-old female patient who presented for consultation after detecting anterior cervical asymmetry and increased volume via self-palpation. The biochemical profile revealed a state of clinical euthyroidism. Cervical ultrasonography demonstrated the absence of thyroid tissue in the right bed and a compensatory left lobe with a volume of 9.5 grams, alongside the presence of a 36 mm dominant nodule (Thyroid Imaging Reporting and Data System (TI-RADS) 4A). The finding of agenesis was incidentally corroborated by cervical magnetic resonance imaging, which also ruled out ectopic tissue. Management consisted of clinical observation and close sonographic surveillance following three fine-needle aspiration biopsies with non-diagnostic results (Bethesda Category I). This case illustrates the relevance of including this dysgenesis in the differential diagnosis of asymmetric cervical masses. Recognizing this anomaly is crucial to avoid unnecessary surgical interventions, such as a diagnostic hemithyroidectomy, or erroneous diagnoses of neoplasia when evaluating single functioning lobes. Conservative management should be prioritized in asymptomatic patients with unilateral agenesis to prevent permanent iatrogenic hypothyroidism. Furthermore, the importance of long-term surveillance is highlighted due to the susceptibility of the single lobe to develop nodular changes and functional exhaustion from chronic hyperstimulation.

## Introduction

The thyroid gland is the first endocrine gland to appear during embryogenesis [1]. Thyroid hemiagenesis (TH) represents a rare congenital disorder characterized by the complete absence of one thyroid lobe, classified within the spectrum of thyroid dysgenesis [2]. Embryologically, lobar agenesis results from an early interruption in the formation or migration of the lateral anlage [3]. Although most cases are sporadic, TH suggests the possible involvement of essential transcription factors, such as PAX-8, TTF-1, and thyroid-stimulating hormone receptor (TSHR) [4,5].

Epidemiologically, TH is a low-frequency entity with an estimated prevalence of approximately 0.2% in pediatric screening studies [6], though its true incidence is likely underestimated due to delayed incidental diagnoses [6,7]. There is a marked asymmetry in its presentation: agenesis of the left lobe occurs in nearly 80% of cases, establishing a left-to-right ratio of 4:1 [8,9]. Therefore, right thyroid lobe agenesis is exceptionally uncommon [8].

The clinical relevance of TH lies in the pathophysiological response of the remnant tissue. Chronic hyperstimulation by thyroid-stimulating hormone (TSH) frequently drives compensatory hypertrophy in the contralateral lobe [7-11]. While many patients remain euthyroid, they often maintain higher TSH concentrations than those with intact thyroids [7,12]. This chronic stimulation acts as a potent mitogenic factor on the single functioning lobe [10,13]. Consequently, there is a higher long-term risk of developing structural pathologies, such as multinodular goiter or autonomous nodules, and a predisposition to hypothyroidism [5,11-16].

Diagnosing congenital TH is crucial to differentiate it from acquired thyroid atrophy [17]. Recognizing this rare right-sided variant is fundamental to avoid diagnostic errors that could lead to unnecessary surgical interventions [18]. This report aims to describe the clinical and imaging findings of right thyroid lobe agenesis with contralateral compensatory hypertrophy. By doing so, it addresses a specific knowledge gap in the current literature: the clinical challenge and bioethical dilemma of managing a highly suspicious, potentially malignant nodule located within a patient's solitary functioning thyroid lobe.

## Case presentation

A 52-year-old female patient from Aguascalientes, Mexico, presented with right thyroid lobe hemiagenesis, compensatory hypertrophy of the left thyroid lobe, and a left thyroid nodule with cystic degeneration (Thyroid Imaging Reporting and Data System (TI-RADS) 4A).

Her family history was significant for a father diagnosed with hyperthyroidism and a sister with a reported atrophy of one thyroid lobe. Her past medical history was unremarkable regarding chronic degenerative diseases, allergies, trauma, or transfusions. She reported occasional alcohol consumption and no smoking history. Her obstetric history was gravida 5, para 4, abortus 1 (G5P4A1). Previous surgical history included a childhood tonsillectomy and a bilateral tubal occlusion, both without complications. Currently, she consumes omega-3 and magnesium supplements.

The condition began insidiously when the patient noticed an increase in volume in the left anterior cervical region through self-palpation, which was accompanied by intermittent cervicalgia. The patient remained completely free of thyroid dysfunction symptoms at all times and denied any compressive symptoms such as dysphagia, dysphonia, or dyspnea. Physical examination revealed an awake, cooperative patient with no characteristic facies. Neck examination showed asymmetry due to left-sided volume enlargement. Palpation delimited a soft, mobile mass dependent on the left thyroid lobe that moved with swallowing. No adenopathies were palpable in the adjacent cervical lymph node chains, and the patient reported no pain upon palpation.

Due to the palpable cervical mass, a cervical ultrasound was performed in February 2025, which reported right thyroid lobe agenesis and sonographic features suggestive of a nodule versus goiter in the contralateral lobe. Three months after symptom onset, laboratory tests reported a thyroid profile within euthyroid ranges (Table 1). Thyroid autoantibodies (anti-TPO and anti-Tg) were not evaluated at the time of the initial assessment. While this represents a limitation in definitively excluding late-stage acquired atrophy, the congenital nature of the agenesis is strongly supported by the imaging findings.

**Table 1:** Patient's thyroid function test results. TSH: Thyroid-stimulating hormone; Free T4: Free thyroxine; Total T3: Total triiodothyronine; Total T4: Total thyroxine.

Parameter	Result	Reference Range
Thyroid-stimulating hormone (TSH)	0.688 µIU/mL	0.35 – 4.94 µIU/mL
Free thyroxine (Free T4)	1.03 ng/dL	0.700 – 1.480 ng/dL
Total triiodothyronine (Total T3)	1.34 ng/mL	0.64 – 1.52 ng/mL
Total thyroxine (Total T4)	6.60 µg/dL	4.8 – 11.72 µg/dL

**Figure 1:**
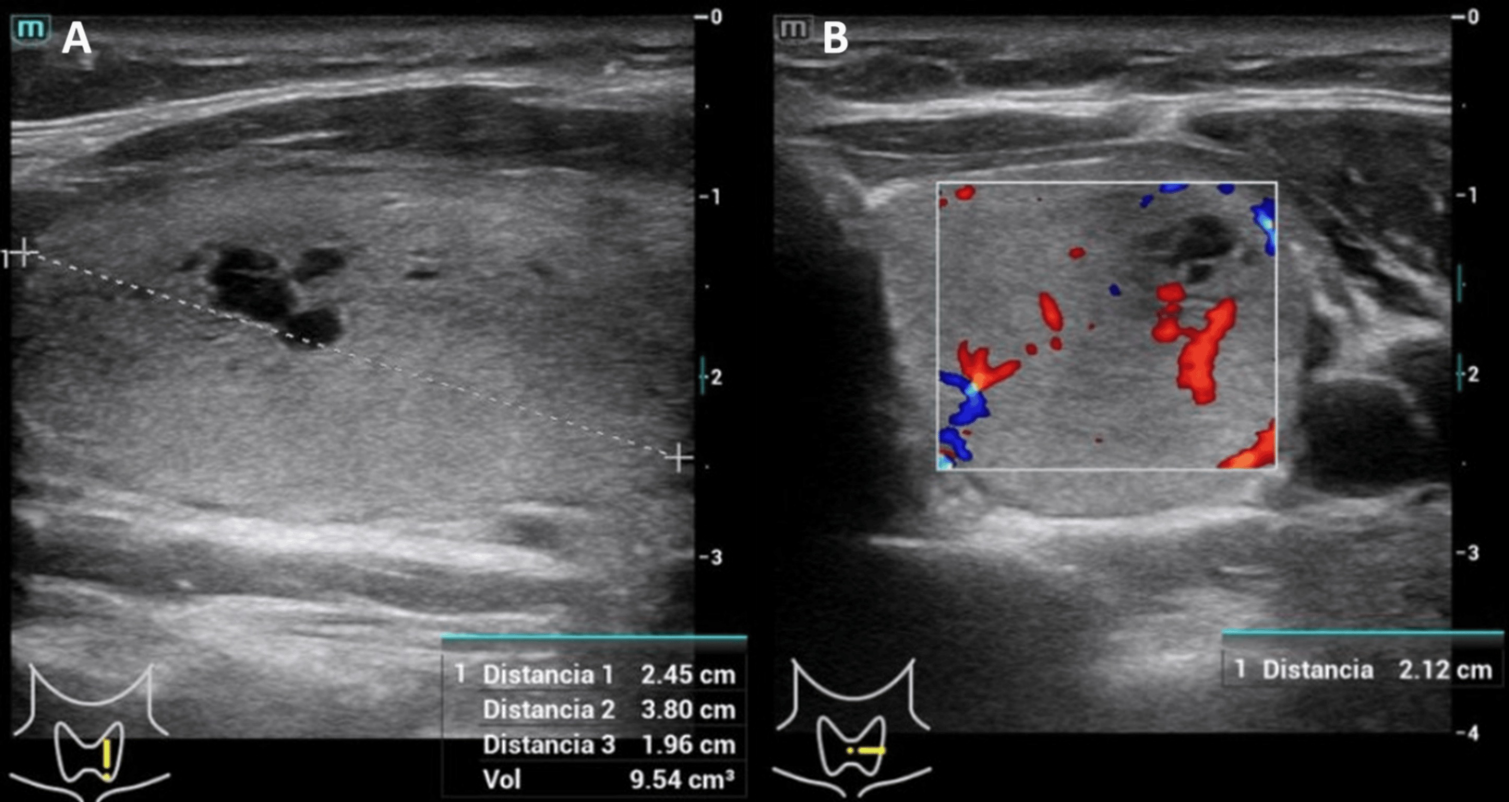
Thyroid ultrasound Thyroid ultrasound. (A) Compensatory hypertrophy of the left lobe (9.5 g) containing a 21 x 36 mm dominant nodule with central cystic degeneration. (B) Color Doppler showing discrete vascularity (TI-RADS 4A). TI-RADS: Thyroid Imaging Reporting and Data System

A subsequent follow-up ultrasound confirmed the absence of the right lobe. Only the left lobe was visible, showing frank compensatory hypertrophy with a volume of 9.5 grams and a heterogeneous echotexture. A large dominant nodule occupying almost the entire lobe was documented, measuring 21 x 36 mm, with circumscribed borders, a discrete halo, and a center with irregular fluid zones indicating probable necrosis or cystic degeneration. It exhibited discrete vascularity and was classified as TI-RADS 4A (Figures 1A, 1B).

**Figure 2:**
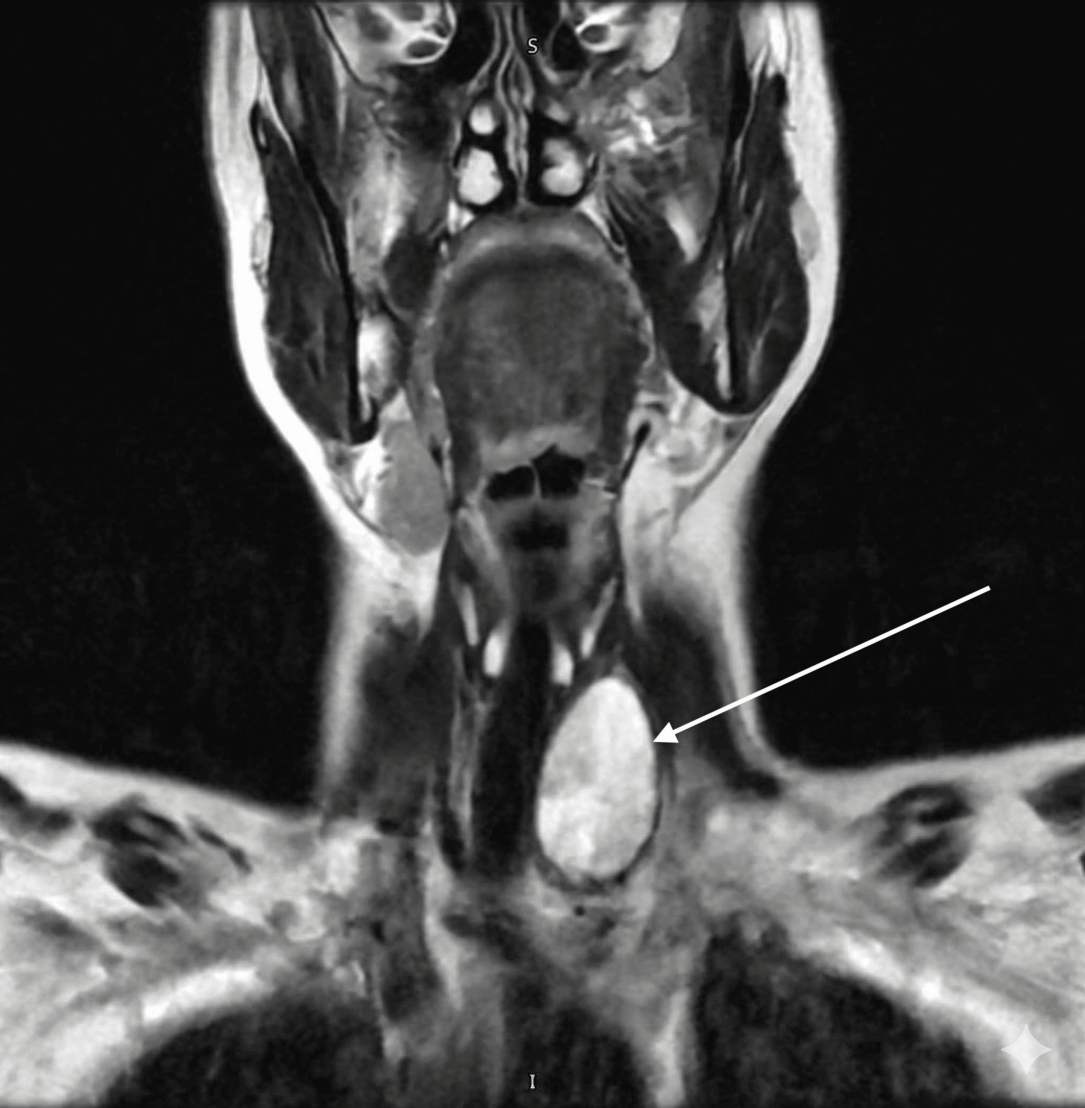
Cervical magnetic resonance imaging, T2-weighted coronal sequence. Cervical MRI, T2-weighted coronal sequence. The arrow indicates massive compensatory hypertrophy of the left lobe assuming total glandular function. Note the complete absence of the right thyroid lobe.

**Figure 3:**
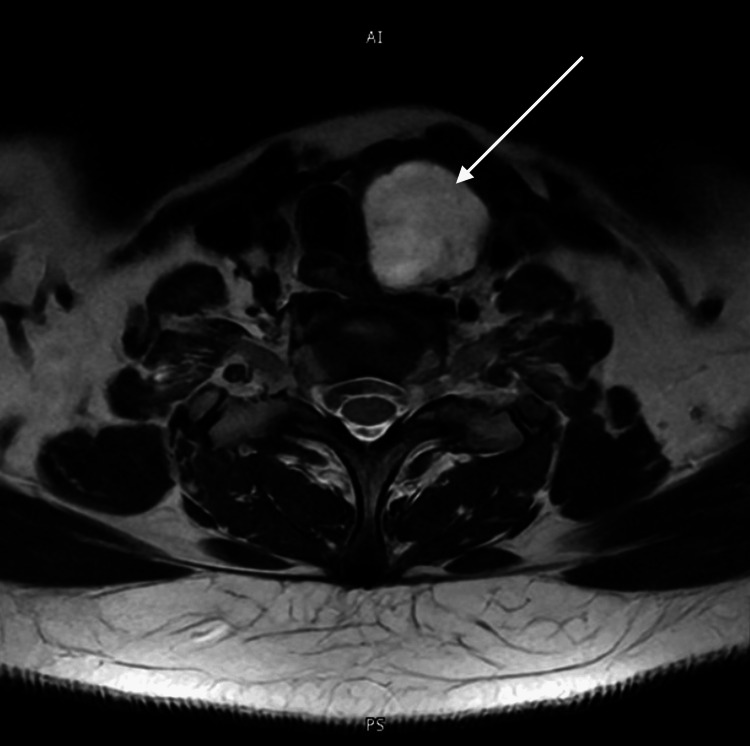
Cervical magnetic resonance imaging, T2-weighted axial sequence. Cervical MRI, T2-weighted axial sequence. The arrow highlights the hyperintense dominant nodule in the left lobe, indicative of cystic necrosis. The empty right thyroid bed is occupied solely by adipose tissue.

Due to the history of cervicalgia and probable left C5 radiculopathy, a non-contrast cervical spine magnetic resonance imaging (MRI) was performed. The images incidentally corroborated the absence of tissue in the right thyroid bed, confirming the hemiagenesis (Figures 2, 3). 

**Figure 4:**
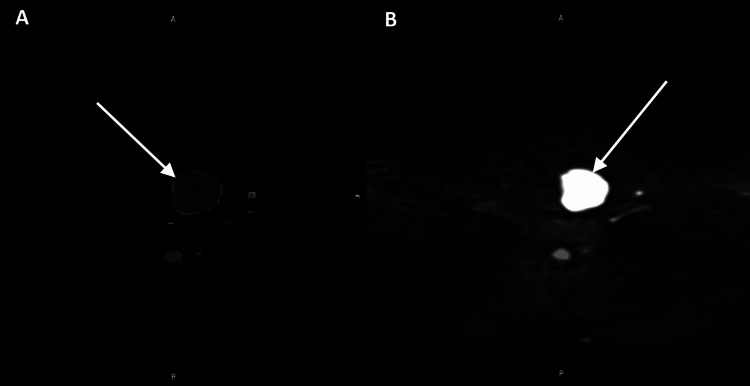
Diffusion-weighted imaging (DWI) (A) and apparent diffusion coefficient (ADC) map (B). The lesion exhibits restricted diffusion (arrows), indicating viscous/proteinaceous content. This key finding explains the repeated nondiagnostic (Bethesda I) FNAB results. FNAB: Fine-needle aspiration biopsy

Following the initial imaging findings, a histopathological study protocol was indicated to evaluate the 36 mm TI-RADS 4A nodule. Over the course of the following months, a total of three sequential fine-needle aspiration biopsies (FNABs) were performed (April, August, and November 2025). In all three procedures, the histopathological reports concluded with a Bethesda Category I result (nondiagnostic or unsatisfactory), obtaining fewer than 15 slides without relevant cellular findings. This is consistent with the extensive cystic degeneration and fluid zones reported on the ultrasound (Figures 4A, 4B).

As the patient was euthyroid with an absolute absence of compressive symptoms, and following three non-diagnostic biopsy attempts due to the nature of the lesion, management was oriented toward preserving thyroid function. A conservative approach was jointly decided. The patient remains stable, euthyroid, and completely asymptomatic. Outpatient management will continue with close clinical and sonographic surveillance of the left thyroid nodule, avoiding unnecessary surgical interventions at this time.

## Discussion

TH represents a congenital disorder of exceptional rarity within the spectrum of thyroid dysgenesis, which constitutes the primary etiology of congenital hypothyroidism [1,8]. From an embryological perspective, lobar agenesis results from an early interruption in organogenesis, specifically a failure in the development or migration of the lateral anlage from the pharyngeal endoderm, a process involving complex developmental signaling pathways [9,16]. Epidemiologically, ultrasound screening studies in asymptomatic populations place its prevalence at merely 0.05% to 0.2% [1,3]. The core atypia of this case lies in its anatomical presentation. The literature documents an overwhelming morphological bias: developmental failure affects the left lobe in nearly 80% of reports, establishing a left-to-right ratio of 4:1 [4,7]. Consequently, isolated right thyroid lobe agenesis, as documented in our patient, constitutes an atypical and exceptionally infrequent clinical variant [4,15].

The natural history of TH is marked by the functional adaptation of the remnant tissue. Physiologically, the absence of 50% of the active glandular mass triggers a pituitary feedback loop that frequently elevates TSH levels [2,6]. This hyperstimulation assumes a role as a potent mitogenic factor, triggering a compensatory hypertrophy of the contralateral lobe, as evidenced in the massive left lobe of our patient [6,10]. Large cohort studies have demonstrated that this vicarious lobe has a significantly increased risk of developing long-term structural pathology, including multinodular goiter, cystic degeneration, and even concurrent autoimmune diseases such as Graves' disease [11,14]. It is fascinating that, despite the frank nodulogenesis and massive hypertrophy, the patient maintains clinical euthyroidism. However, the TSH level of 0.688 mIU/L, while within the reference range, is at the lower end of the spectrum. In the context of a single functioning lobe where high-normal or elevated TSH levels are typically expected to drive compensatory hypertrophy, this low-normal value provides a crucial insight into the nodule's pathogenesis. It strongly suggests an evolving functional autonomy within the 36 mm macronodule. As the nodule grows, it likely acquires autonomous hormone production driven by local autocrine growth factors or somatic mutations, becoming independent of the TSH stimulus [5,15]. Consequently, this autonomous micro-secretion exerts early negative feedback on the pituitary gland, progressively suppressing TSH levels. This transition from TSH-dependent compensatory hypertrophy to TSH-independent nodular autonomy highlights the susceptibility of the remnant lobe to progress toward subclinical hyperthyroidism over time.

The clinical approach to unilateral asymmetrical goiter requires a meticulous differential diagnosis to distinguish it from late-acquired thyroid atrophy [17]. Ultrasound is positioned as the initial gold standard [3,17]. In our case, it revealed a 36 mm dominant nodule classified as TI-RADS 4A, making it necessary to rule out malignant pathology, since dysgenesis does not exempt the development of well-differentiated thyroid carcinomas [12]. Nevertheless, the medical team faced a profound diagnostic challenge: obtaining three FNABs with Bethesda Category I (nondiagnostic) cytology. This discordance does not represent a technical failure but is fully consistent with the extensive cystic and necrotic degeneration of the nodule described on imaging. In this scenario, the cervical magnetic resonance imaging, initially indicated for suspected C5 radiculopathy, played an incidental but defining role: it confirmed the empty right thyroid bed and conclusively ruled out the presence of ectopic tissue, an unavoidable step in the characterization of dysgenesis [15].

Although TH has historically been considered a sporadic event, recent genomic evidence underscores the involvement of mutations in thyroid transcription factors (TTF-1, TTF-2, PAX-8) and in the TSHR [2,8,9]. An invaluable contribution of this report to the body of literature is the detailed family pedigree of the patient: a father with hyperthyroidism and a sister with a history of "atrophy" of one thyroid lobe. While this familial cluster is intriguing, it remains an observational finding and must be interpreted cautiously. The "atrophy" reported by the sister could theoretically represent an undiagnosed hemiagenesis or severe autoimmunity. This familial aggregation raises the hypothesis of a potential hereditary transmission of susceptibility to thyroid dysgenesis. Although speculative in the context of a single case report, it echoes known genetic findings and highlights the need for further genomic research in familial cohorts to substantiate these associations [2,8].

According to the American Thyroid Association (ATA) guidelines, a repeat ultrasound-guided FNAB is recommended for initially nondiagnostic nodules to achieve a definitive diagnosis. The clinical decision to pursue three sequential FNABs in our patient was strictly driven by this guideline-based rationale, aiming to definitively rule out malignancy in a 36 mm TI-RADS 4A nodule without immediately resorting to surgery. However, as the repeated cytologies persistently yielded nondiagnostic results (Bethesda I), and from an algorithmic standpoint, this usually leads to a diagnostic hemithyroidectomy [12], the medical team faced a severe bioethical dilemma. However, in a patient with hemiagenesis, this traditional approach triggers a severe bioethical dilemma. The resection of the left lobe would have entailed the extirpation of 100% of the thyroid functional reserve, condemning the patient to permanent and chronic iatrogenic hypothyroidism, with all the systemic and metabolic implications that hormonal deprivation entails [18]. Appealing to the guiding principle of non-maleficence (primum non nocere), the clinical decision to institute conservative management is incontrovertible. Given a state of sustained euthyroidism, the predominantly cystic nature of the lesion, and the absolute absence of compressive symptoms, organ preservation is a priority [13,15]. A long-term sonographic and biochemical surveillance protocol has been established, a strategy that mitigates surgical risk without neglecting the oncological and functional monitoring of the only remnant tissue [11,15].

## Conclusions

Right thyroid lobe agenesis constitutes an embryonic dysgenesis of exceptional rarity that breaks the usual epidemiological paradigm of thyroid asymmetries. This report conclusively demonstrates that compensatory hypertrophy of the contralateral lobe, as a functional adaptation mechanism, can evolve toward structurally suspicious macronodular degeneration (TI-RADS 4A) even in the presence of strict clinical and biochemical euthyroidism. The greatest diagnostic and therapeutic challenge in these scenarios is radiopathological discordance. In the face of repeatedly nondiagnostic cytological findings (Bethesda Category I) secondary to extensive necrosis or cystic degeneration, comprehensive evaluation supported by cross-sectional imaging (such as magnetic resonance imaging) is crucial to rule out ectopic tissue and confirm true agenesis.

From a clinical and bioethical perspective, this case offers a valuable case-based insight for surgical and endocrinological practice: when faced with an asymptomatic asymmetric cervical mass in the context of hemiagenesis, conservative management should be strongly considered as a preferable alternative to an immediate diagnostic hemithyroidectomy. Avoiding the extirpation of the only remaining functional tissue honors the principle of non-maleficence, preventing irreversible iatrogenic hypothyroidism. Finally, the strong cluster of thyroid pathology identified in the patient's family history highlights the imperative need to consider a genetic background in dysgenesis, making it mandatory to establish a lifelong sonographic and biochemical surveillance protocol to preserve organ function and the patient's quality of life.

## References

[1] Maiorana R, Carta A, Floriddia G (2003). Thyroid hemiagenesis: prevalence in normal children and effect on thyroid function. J Clin Endocrinol Metab.

[2] Szczepanek-Parulska E, Zybek-Kocik A, Wartofsky L, Ruchala M (2017). Thyroid hemiagenesis: incidence, clinical significance, and genetic background. J Clin Endocrinol Metab.

[3] Shabana W, Delange F, Freson M, Osteaux M, De Schepper J (2000). Prevalence of thyroid hemiagenesis: ultrasound screening in normal children. Eur J Pediatr.

[4] Tiwari PK, Baxi M, Baxi J, Koirala D (2008). Right-sided hemiagenesis of the thyroid lobe and isthmus: a case report. Indian J Radiol Imaging.

[5] Briñez AM, Núñez JM, Suárez CM, Andreina Andreina (2021). Thyroid hemiagenesis: clinical case and literature review (Article in Spanish). Rev Venez Oncol.

[6] Unar AA, Akhtar S, Ghaloo SK, Awan MO, Anjum S (2024). Thyroid hemiagenesis with compensatory hypertrophy of the remaining lobe: a case report. J Pak Med Assoc.

[7] Alcón Sáez JJ, Yeste Fernández D, Elía Martínez MA, Gussinyé Canadell M, Carrascosa Lezcano A, Goya E (2005). Thyroid hemiagenesia diagnosed in a 5-month-old infant (Article in Spanish). An Pediatr (Barc).

[8] Park SM, Chatterjee VK (2005). Genetics of congenital hypothyroidism. J Med Genet.

[9] De Felice M, Di Lauro R (2004). Thyroid development and its disorders: genetics and molecular mechanisms. Endocr Rev.

[10] Segura SS, Jiménez JQ, de Escobar GM (2009). Frequent thyroid diseases in childhood (Article in Spanish). Rev Pediatr Aten Primaria.

[11] Ruchala M, Szczepanek E, Szaflarski W (2010). Increased risk of thyroid pathology in patients with thyroid hemiagenesis: results of a large cohort case-control study. Eur J Endocrinol.

[12] Martín JG, Rizo SE (2016). Current aspects of well-differentiated thyroid carcinoma (Article in Spanish). Rev Cubana Cir.

[13] McHenry CR, Walfish PG, Rosen IB, Lawrence AM, Paloyan E (1995). Congenital thyroid hemiagenesis. Am Surg.

[14] Benito IH, Sánchez AR, Aznar DL (2001). Association of thyroid hemiagenesis and Graves' disease (Article in Spanish). Rev Esp Med Nucl.

[15] Elorza DJ, Rossat AC, Orellana EO (2010). Right lobe thyroid hemiagenesis: a case report (Article in Spanish). Rev Chil Cir.

[16] Shanmugalingam S, Houart C, Picker A (2000). Ace/Fgf8 is required for forebrain commissure formation and patterning of the telencephalon. Development.

[17] Mikosch P, Gallowitsch HJ, Kresnik E, Molnar M, Gomez I, Lind P (1999). Thyroid hemiagenesis in an endemic goiter area diagnosed by ultrasonography: report of sixteen patients. Thyroid.

[18] Pringle PJ, Stanhope R, Hindmarsh P, Brook CG (1988). Abnormal pubertal development in primary hypothyroidism. Clin Endocrinol (Oxf).

